# Gambling, Gaming, and Internet Behavior in a Sexual Minority Perspective. A Cross-Sectional Study in Seven European Countries

**DOI:** 10.3389/fpsyg.2021.707645

**Published:** 2022-04-11

**Authors:** Niroshani Broman, Fulvia Prever, Ester di Giacomo, Susana Jiménez-Murcia, Anna Szczegielniak, Helena Hansson, Anders Håkansson

**Affiliations:** ^1^Department of Clinical Sciences Lund, Faculty of Medicine, Lund University, Lund, Sweden; ^2^Gambling Disorder Unit, Malmö Addiction Center, Region Skåne, Malmö, Sweden; ^3^National Health System (NHS), Addiction Department, Milan, Italy; ^4^SUN(N)COOP Scientific Director “Women and Gambling Project,” Milan, Italy; ^5^Section of Forensic Psychiatry, King’s College London, Institute of Psychiatry, London, United Kingdom; ^6^School of Medicine and Surgery, University of Milan Bicocca, Milan, Italy; ^7^Psychiatric Department -Azienda Socio-Sanitaria Territoriale (ASST), Monza, Italy; ^8^Department of Psychiatry, Bellvitge University Hospital-IDIBELL, Barcelona, Spain; ^9^Ciber Fisiopatología Obesidad y Nutrición (CIBERObn), Instituto de Salud Carlos III, Madrid, Spain; ^10^Department of Clinical Sciences, Faculty of Medicine, University of Barcelona, Barcelona, Spain; ^11^Faculty of Medical Sciences in Katowice, Medical University of Silesia, Katowice, Poland

**Keywords:** gambling, gaming, sexual minorities, Europe, lesbian, gay, bisexual, internet behavior

## Abstract

**Background:**

Addictive behavior of gambling, gaming and internet activity is partly a new research domain and has not been well investigated with regard to sexual minority populations. Although health disparities between sexual minorities and the general population are well documented, there is a lack of inclusion of sexual minorities in both research and clinic. Among lesbian, gay and bisexual populations certain features could be present that play a role for the development of addictive behaviors, such as social isolation and increased risk of other psychiatric problems. The aim of this study was to investigate problem gambling, problem gaming and problematic internet behavior in a European context and if it is affected by sexual orientation status.

**Methods:**

An online web-survey was distributed among web-panels in England, Poland, Switzerland, Italy, Spain, Denmark, and Sweden in 2017–2018.

**Result:**

10 983 complete answers were collected. 7.1% of the participants had a sexual minority status (*n* = 774). Regression models found that there was no difference in gambling, gaming and internet behavior among heterosexual and sexual minority men. Sexual minority women were associated with problematic gambling and gaming behavior, when also controlling for age and nationality. When also controlling for psychological distress, women defining as having another sexual minority status than lesbian and bisexual remained significant for having a problematic gaming behavior (AOR = 2.3).

**Conclusion:**

An awareness of female sexual minority perspectives is relevant in facilities treating behavioral addiction as well as in future research in behavioral addiction. More research is needed in problematic gambling and gaming behavior in different sexual minority populations with regard to psychiatric comorbidity and living conditions. An inclusion of sexual minority groups defining as other than gay and bisexual is needed in future research. No significant differences were found between heterosexual and sexual minority men in adjusted analysis in this study.

## Background

Gambling with money is an activity that for some individuals could lead to a problematic behavior, with different levels of severity. Pathological forms of gambling have shown great similarities with substance dependence in literature with a development of a tolerance, withdrawal symptoms, failed efforts to stop gambling and severe social and relational consequences, according to the American Psychiatric Association (American Psychiatric Association [APA], 2013). Despite the similarities in gambling disorder and substance dependence, it was not until 2013 that pathological gambling was moved to the chapter of addiction disorders in the DSM-5 manual (American Psychiatric Association [APA], 2013). Earlier, it had been categorized as an impulse control disorder. Gambling disorder has not been as well documented as other addiction disorders in a global perspective, although it has been associated with a difficult psycho-social and socio-economic situation (Muggleton et al., 2021), a high comorbidity with other psychiatric conditions (Castrén et al., 2013; Dowling et al., 2015; Håkansson et al., 2018) and suicidal behavior (Black et al., 2015), including completed suicide (Karlsson and Håkansson, 2018). Past-year prevalence of any kind of gambling problem is varying between 0.1 and 5.8% globally in an overview article by Calado and Griffiths (2016). Gambling problems have been associated with a lower degree of education, male gender and a risky alcohol consumption (Wong et al., 2003; Abbott et al., 2014; Del Pino-Gutiérrez et al., 2017). Globally, gambling problems among women seem to have a later onset than among men, and may be associated with a higher psychiatric comorbidity than in men with gambling problems (Díez et al., 2014; Carneiro et al., 2019). Gambling is an activity that in some settings is showing a rapid movement from mainly land-based gambling forms, into online forms such as casinos and online betting forms (McCormack et al., 2013; Håkansson et al., 2017). Online gambling could contain certain features that are suggested to increase the risk of developing a pathological gambling, and a regular participation in online casino and betting has been correlated with a higher rate of gambling problems (McCormack et al., 2013).

The most recent addiction disorder in ICD-11 (World Health Organization [WHO], 2018) is the internet gaming disorder, i.e., a pathological use of electronical games which is still a tentative diagnosis in DSM-5 (American Psychiatric Association [APA], 2013), due to an insufficient amount of research at the time. Playing electronical games is a common activity among adolescents and young adults, that could be related to positive features such as improved short-term memory (Adachi and Willoughby, 2013). For the large majority of users, it is a leisure activity with no relation to a problematic behavior. A low to moderate participation in electronical games (1–5 h per week) has even been associated with lower substance usage than among non-players in a recent publication (Turel and Bechara, 2019). A qualitative interview study also supports the theory that electronical games could be protective factor against substance use among adolescents (Törrönen et al., 2019). Bearing this knowledge in mind, it still seems as for a minor part of the population the gaming activity has been suggested to develop into an addictive behavior. The prevalence of gaming disorder is suggested to range from 1.2 to 1.7% among adolescents in European contexts (Rehbein et al., 2015; Vadlin et al., 2015; Wichstrøm et al., 2019). Excessive gaming involves spending a large amount of time on gaming, loss of control, aggressive behavior, conflicts with family members and withdrawal symptoms, related to sudden stops in gaming activity (Lemmens et al., 2009; Gentile et al., 2017). Individuals with gaming problems have been associated with personal traits that could be a challenge for establishing “real-life” relations, such as neuroticism, shyness and low self-esteem (Peters and Malesky, 2008; van Rooij et al., 2014; Laconi et al., 2017). Though gaming could involve both offline and online games, specific types of online games have been associated with gaming problems to a higher degree than offline games. Massive multiplayer online role-playing games (MMORPG) are the type of games that often are mentioned, where the players interact with each other during the game session (Peters and Malesky, 2008; American Psychiatric Association [APA], 2013). Given the fast development of the gaming culture and multiplayer online games, the research within the field is still scarce. As with gambling disorder and substance-related disorders, gaming problems seem to be overrepresented among men in the general population (Lemmens et al., 2015; Vadlin et al., 2016).

Research in behavioral addictions is still scarce and the area is somewhat controversial. There is an ongoing dialogue on which behaviors that might be included in the future where social media and other electronical activities than games and potential health effects are discussed (Kuss and Griffiths, 2011; Yau and Potenza, 2015; Chamberlain et al., 2016; Grant et al., 2017). Although it seems as it is the kind of online activity that could be problematic and in some cases addictive (Young, 1999; Kuss and Griffiths, 2011), an excessive use of the internet in general has been related to a poorer mental health status. A problematic use of the internet could be defined as an excessive use leading to emotional, physical, social or functional impairment (Moreno et al., 2016). In a publication exploring addictive internet behavior in a psychiatric population, excessive internet usage was 3.29 times higher in the group with psychiatric conditions compared to the control group (Yar et al., 2019).

Prevalence of mental health problems, suicidal thoughts and behavior and substance use problems have been more extensive among lesbian, gay and bisexual individuals, compared to heterosexual groups (Cochran et al., 2004; Marshal et al., 2009; Corliss et al., 2010; Lucassen et al., 2017; Di Giacomo et al., 2018). Substance use problems seem to have an earlier age of onset than among heterosexual youth and persist into adulthood in a larger extent (Marshal et al., 2009; Dermody et al., 2014; Schuler and Collins, 2020). Most research is conducted in Northern American settings, although the same pattern in health disparities for substance use and mental health problems has been demonstrated for sexual minorities in Europe in studies and reports (Russell and Fish, 2016; Björkenstam et al., 2017; Bränström and Pachankis, 2018; Di Giacomo et al., 2018). Substance use problems have been more prevalent among lesbian and bisexual women compared to heterosexual women in several studies (Roxburgh et al., 2016; Drabble et al., 2018; Schuler and Collins, 2020). Bisexual women might have a higher risk for substance use problems compared to lesbian women in some settings (Schuler and Collins, 2020).

Research on gambling and gaming activities in among lesbian/gay, bisexual and other sexual minority populations is scarce. Two studies have examined gambling behavior and sexual orientation in Northern American settings, where of both studies revealed a higher prevalence of gambling problems among sexual minorities (Grant and Potenza, 2006; Richard et al., 2019). For gaming, previous literature is lacking, but a few publications have shown a higher prevalence of gaming problems among sexual minority populations, including a pilot project to this study in a Swedish setting (Broman and Håkansson, 2018). Historically, the LGBTQ-population has faced several challenges considering how sexual and gender minorities have been criminalized, considered perverted and pathologized (Drescher, 2015). Living conditions still differ immensely in different European settings (European Union Agency for Fundamental Rights [FRA], 2013), reasonably affecting the willingness of openness in different settings. Although health disparities between sexual and gender minorities and the general population are well documented, there is still a lack of inclusion of these minority groups within research concerning medicine and health.

The minority stress theory (Meyer, 2003) is a theoretical framework for understanding how stigma due to sexual or gender minority status could explain the disparities in health between the LGBT-population and the general population. Living conditions for the LGBTQ-population and the general population might impact the degree of openness and trust for health care system and social services (Röndahl, 2009; Ramos et al., 2019). The living conditions also seem to differ depending on the sexual orientation status. Discrimination and experience of psychological and physical violence has been more frequently reported among the lesbian, gay, and bisexual populations than in the general population in several settings (Blosnich et al., 2015; Bränström, 2017; McKay et al., 2017), which supports the theory that the disparities in health could be a result of minority stress. In some settings, homosexual and bisexual women more frequently report experiences of stigma and victimization and more mental health problems compared to heterosexual women (Hequembourg and Brallier, 2009; Roxburgh et al., 2016; Björkenstam et al., 2017). Bisexual women have also presented more substance-related problems and psychological distress compared to heterosexual women (Bränström and Pachankis, 2018). A number of publications have shown an association between stigma in forms of discrimination, violence, less satisfying economic and social features and a higher risk for mental health and substance use problems among lesbian, gay and bisexual populations (Lehavot and Simoni, 2011; Hatzenbuehler and Pachankis, 2016; Gustafsson et al., 2017; Bränström and Pachankis, 2018; Bränström et al., 2020).

The health disparities between these groups and the general population are well documented; yet, sexual minorities are typically reached to a lesser extent than others by clinical interventions and in research (Ash and Mackereth, 2013; Russell and Fish, 2016). Among sexual minorities certain features could be present that play a role for the development of addictive behaviors, such as social isolation and increased risk of other psychiatric problems. Based on these knowledges, and due to the fact that behavioral addiction is still a young research field, the aim of this study was to analyze whether problem gambling, problem gaming and problematic internet behavior are more common in individuals belonging to sexual minorities in a number of European countries.

## Materials and Methods

The investigation was designed as a web survey and was performed in seven European countries; Sweden, Denmark, England, Spain, Italy, Poland, and Switzerland. All data was self-reported. The investigation was carried out between August 2017 and June 2018. The researchers cooperated with a company called Userneeds.^1^ Userneeds is a company operating in several European countries, providing web panels of voluntary online users that have agreed to participate in different types of surveys, normally within marketing research. The participants in the web panels are given credits for completing a survey, based upon the time it takes to fill in the questionnaire. The credit had a monetary value, which in this survey corresponded to 1 euro. Userneeds provided the researchers with approximately 1,500 complete answers in each country, representable for age and biological gender compared to the general population in each country or province. All participants had to fill out an electronical informed consent, by clicking a box where they confirmed to be > 15 years of age and that they had understood the information, before gaining access to the survey. Fifteen years of age was the age limit for when a parental consent was not needed for participation in most included countries. The exceptions were England and Poland, where the age limit for participation without parental consent was 16 years and Italy, where 18 years of age was the legal age for participation. No information was gathered about names, social security number or geographical whereabouts that made the participants identifiable by any of the companies or the researchers. IP-addresses were not accessible for the researchers. In order to complete the survey, all questions were decided to be mandatory. Ethical approval was collected from the regional ethics committee from each country where this was required (Sweden, Italy, Poland and Spain). A direct dialogue was held with the regional ethics committee in Denmark, England and Switzerland who informed the researchers that the survey was not considered to require an approval from them.

### Instrument and Measures

All measures in this investigation were self-reported. Sociodemographic variables collected in the survey were age, occupation, sexual orientation (heterosexual, homosexual, bisexual and other) and gender (male, female, transgender). Apart from the three instruments screening for behavioral addictions and sociodemographic variables, three additional questions were asked. The first question aimed to describe social isolation, asking whether the respondents had a satisfying number of social contacts (outside the internet), had too many social contacts, or felt lonely and wished to have more social contacts. The participants were then asked if they had ever felt the need to seek professional help due to psychological distress, a question that was optional (the alternative “I do not wish to answer” was added). Finally, the survey contained a question investigating how many hours that was normally spent communicating with others online per day, including social medias, WhatsApp, Skype, online rooms (including chat rooms in games). The response alternatives were less than 1, 1–2, 2–3, 3–4, or 4 h or more, where 3–4 h or more was used as a cut-off indicating a high use of online communications. Sexual orientation was categorized into two groups (heterosexual vs. homosexual, bisexual and other). All variables were dichotomized for the bivariate analysis. Gender was categorized into male and female. Due to requirements from the regional ethics committee in Italy, sexual orientation was optional in the Italian survey (“I prefer not to say was added as response alternative”). Apart from the question about sexual orientation in the Italian survey and the question about psychological distress (in all countries), remaining questions were mandatory in order to complete the survey. Since the number of transgender participants were few in the whole material (*n* = 16), they were excluded from the analyses. The Italian respondents who chose not to reveal their sexual orientation (*n* = 21), and answers that were partial, were also excluded from the final analysis (*n* = 966).

The three following instruments were used to assess the three different addictive behaviors. A back-translation into English, carried out by an independent person with native level of Swedish and English, was judged to yield satisfactory results.

1. The NODS-CLiP screening instrument, consisting of 3 questions with a “yes” or “no” response alternative, was used to assess gambling problems. The cut-off used for gambling problems, was one point or more on the NODS-CliP instrument. The instrument is suitable for epidemiological population surveys as well as in clinical settings, and with a documented high sensitivity and specificity (Toce-Gerstein et al., 2009). Internal consistency was calculated with Cronbach’s alpha value of 0.68.
2. Gaming problems were identified using the game addiction scale (GAS) for adolescents. The instrument consisted of a Likert scale with 1–5 points on each of the 7 items, ranging from never to very often. For a problematic gaming behavior, having at least 3 points on 4 of the 7 items was used as a cut-off, in accordance with Lemmens et al. (2009). Internal consistency was calculated with Cronbach’s alpha value of 0.89.
3. The original version of the Problematic and risky internet use screening scale (PRIUSS) contains 18 items. A problematic internet behavior in this survey was assessed with the 3-items PRIUSS Likert scale, a shorter version of the original 18 items scale, ranging from never to very often. The completed score for an individual was 1–12 points and 9 points or more was used as a cut-off for a problematic behavior, the higher cut-off defined by Moreno et al. (2016). Internal consistency was calculated with Cronbach’s alpha value of 0.81.

At the end of the survey, a recommendation was provided to the respondents; risk behavior (and an additional message for higher risk) was communicated, along with a recommendation to seek help, and with a stronger emphasis for participants reaching the higher problem level. The higher problem level for gambling problems was defined as 3 points on the NODS-CLiP instrument, > 3 points on at least 4 items on the GAS-scale for gaming problems, and 9–12 points on the PRIUSS scale for a problematic internet use.

### Statistics

Analyses were performed in SPSS version 24. Chi-square test was used for all categorical variables and bivariate analysis, to describe differences between the groups reaching the threshold for a problematic gambling, gaming or internet behavior with the groups with no problematic behavior. All of the three dependent variables (problematic gambling, gaming and internet behavior) were controlled for age, country affiliation, loneliness and a wish for having more social contacts (outside the internet), having considered seeking professional help due to psychological distress, gender and sexual orientation (a heterosexual group and one minority group) in a binary regression analysis. Applying binary regression analysis, country affiliation was added as an independent, categorical variable. First, analyses were conducted separately for women, and men, respectively. For each of the addictive behaviors assessed (gambling, gaming, internet use), we finally conducted a full logistic regression analysis including all eligible subjects and controlling for gender (male vs. female).

Analyses were performed in the whole material, where all seven countries were included in the same analysis. Due to translation problems in the Polish gambling screening protocol, the Polish gambling result was excluded from the gambling analysis. In the Italian survey, > 50% of the participants fulfilled criteria for gambling problems for reasons that were not evident to the researchers. It was considered to bias the gambling result and the Italian gambling result was excluded from the analysis. A higher proportion than expected chose “other” as occupational status (see Table 1) in the whole material, considered to entail a risk for a bias by the researchers and occupation was excluded from chi square tests and regression analysis. Separate analysis was performed for women and men. Associations with a *p*-value less than 0.05 were considered significant, and the binary regression model was analyzed with 95% confidence intervals (CI) for each of the potential correlates of problem gambling.

**TABLE 1A:** Descriptive data, gender and sexual orientation.

Sexual orientation	All (*N* = 10,983)	Women (*n* = 5,448)	Men (*n* = 5,519)	Transgender (*n* = 16)
Heterosexual	92.9% (*n* = 10,199)	93.2% (*n* = 5,079)	92.7% (*n* = 5,114)	37.5% (*n* = 6)
Sexual minority status	7.1% (*n* = 774)	6.7% (*n* = 366)	7.4% (*n* = 405)	
Homosexual	2.7% (*n* = 294)	1.5% (*n* = 84)	3.8% (*n* = 207)	18.8% (*n* = 3)
Bisexual	2.8% (*n* = 309)	3.5% (*n* = 191)	2.1% (*n* = 117)	6.3% (*n* = 1)
Other	1.6% (*n* = 181)	1.7% (*n* = 94)	1.5% (*n* = 81)	37.5% (*n* = 6)

**TABLE 1B:** Number of individuals with scores above cut-off for problem gambling, problem gaming, and problematic internet use, per country.

	Gambling (NODS-CLiP), above cut-off	Gaming (GAS), above cut-off	Problematic internet use (PRIUSS value)
All countries	18.9% (*n* = 1,508)	12.6% (*n* = 1,385)	3.7% (*n* = 403)
Median value	0	8.0 (IQR 7–13)	2.0 (IQR 0–4)
Sweden	9.9% (*n* = 154)	6.1% (*n* = 95)	2.1% (*n* = 33)
Denmark	12.2% (*n* = 196)	4.1% (n = 66)	1.0% (n = 16)
England	23.9% (*n* = 362)	18.8% (*n* = 285)	5.5% (*n* = 84)
Italy	–	16.1% (*n* = 239)	4.3% (*n* = 63)
Spain	28.7% (*n* = 433)	20.7% (*n* = 313)	5.5% (*n* = 83)
Poland	–	18.4% (*n* = 274)	6.4% (*n* = 97)
Switzerland	20.4% (*n* = 363)	6.1% (*n* = 110)	1.1% (*n* = 28)

**TABLE 1C:** Social isolation and psychological distress in groups with different sexual orientation.

Number of social contacts outside the internet	All	Heterosexual	Gay/lesbian	Bisexual	Other
Would have wished for more, feeling lonely	16.1% (*n* = 1,765)	15.6% (*n* = 1,586)	22.4% (*n* = 66)	24.6% (*n* = 76)	22.1% (*n* = 40)
Satisfactory or too many social contacts	83.9% (*n* = 9,204)	84.4% (*n* = 8,613)	77.6% (*n* = 141)	75.4% (*n* = 233)	77.9% (*n* = 228)
**Ever felt need to seek professional help for psychological distress**					
Yes	22.6% (*n* = 2,481)	21.7% (*n* = 2,211)	33.7% (*n* = 99)	44.3% (*n* = 137)	21% (*n* = 38)
No	77.4% (*n* = 8,488)	78.3% (*n* = 7,988)	66.3% (*n* = 195)	55.7% (*n* = 172)	79% (*n* = 143)

## Results

### Sample Characteristics

A total of 11 955 participants were included, where of 10 983 answers were complete and included in the final analysis. Among these, 16 defined as transgender. No result is presented in the study specifically for the transgender group since it was considered too small to perform any comparing analysis. A total of 392 subjects (3.5 percent) were in the youngest age group (15–18 years), nine percent (*n* = 1,013) were 19–24 years old, 10 percent (*n* = 1,106) 25–29 years, 19 percent (*n* = 2,121) 30–39 years, 20 percent (*n* = 2,266) 40–49 years, 19 percent (*n* = 2,171) were 50–59 years old, and 19 percent (*n* = 2,144) were 60 years of age or older. The socio-demographic characteristics are summarized in Tables 1A–C.

### Result Gambling

In the five included countries (Sweden, Denmark, Switzerland, England, Spain, *n* = 7,982), gambling problems were positively correlated with loneliness (14.5 vs. 19.1% among problem gamblers, *p* < 0.001), psychological distress (22.3 vs. 32.7% among problem gamblers, *p* < 0.001), male gender (14.0% reached the threshold for gambling problems among women vs. 23.8% among men, *p* < 0.001) and having a sexual minority status (18.6% in the heterosexual population fulfilled criteria for gambling problems vs. 23.1% in the minority population, *p* = 0.004).

When analyzing the female population in the five included countries (*n* = 3,949), 14% (*n* = 552) had 1–3 points on the NODS-CLiP instrument, indicating gambling problems. Female problem gamblers were significantly more likely to have experienced loneliness (15.4 vs. 21.6% among problem gamblers, *p* < 0.001), have experienced psychological distress (28.4 vs. 42.9% among problem gamblers, *p <* 0.001) and have a sexual minority status (13.5% in the heterosexual group fulfilled criteria for a gambling problem vs. 20.4% in the minority group *p* = 0.001).

In the male population (*n* = 4,019), 23.8% reached the level for having a gambling problem on the NODS-CLiP instrument. Problem gambling in the male population was associated with loneliness (13.4 vs. 17.8% among problem gamblers, *p* < 0.001) and psychological distress (15.4 vs. 26.9% among problem gamblers, *p* < 0.001). No statistical significance was found for sexual orientation; 23.6% (*n* = 874) reached the threshold for gambling problems in the heterosexual group vs. 26.2% (*n* = 84) in sexual minority group (*p* = 0.164).

In logistic regression, controlling for age, country, social isolation and a need to seek professional help due to psychological distress, a sexual minority status did not have a statistical significance for gambling problems among women (Table 2) or among men (Table 3). When including women and men in a full, adjusted analysis, problem gambling was significantly associated with younger age, social isolation, need to seek treatment, and with male gender, whereas no association was seen with sexual minority status (Table 4).

**TABLE 2:** Problematic gambling women (*n* = 3,948).

	Odds ratio (OR)	Confidence interval (CI)	*p*-value
Sweden	1		
Denmark	1.411	0.947–2.103	0.090
England	3.729	2.620–5.309	<0.001
Spain	6.159	4.352–8.714	<0.001
Switzerland	3.692	2.597–5.247	<0.001
Social isolation	1.245	0.977–1.586	0.076
Experienced need for seeking health care	2.072	1.694–2.533	<0.001
Age	0.869	0.823–0.918	<0.001
Heterosexual	1		
Gay/lesbian	1.338	0.706–2.539	0.372
Bisexual	1.213	0.786–1.874	0.384
Other sexual orientation	1.390	0.750–2.576	0.296

**TABLE 3:** Problematic gambling men (*n* = 4,019).

	Odds ratio (OR)	Confidence interval (CI)	*p*-value
Sweden	1		
Denmark	1.287	0.972–1.705	0.078
England	2.718	2.090–3.535	<0.001
Spain	3.136	2.411–4.079	<0.001
Switzerland	2.356	1.815–3.058	<0.001
Social isolation	1.182	0.954–1.465	0.125
Experienced need for seeking health care	1.897	1.574–2.288	<0.001
Age	0.820	0.783–0.860	<0.001
Heterosexual	1		
Gay/lesbian	0.758	0.517–1.110	0.154
Bisexual	0.903	0.548–1.488	0.689
Other sexual orientation	1.342	0.761–2.366	0.310

**TABLE 4:** Variables potentially associated with problem gambling.

	Odds ratio (OR)	Confidence interval (CI)	*p*-value
Sweden	1		
Denmark	1.351	1.076–1.697	0.010
England	3.070	2.491–3.783	<0.001
Spain	4.140	3.366–5.092	<0.001
Switzerland	2.781	2.258–3.425	<0.001
Male gender	2.375	2.098–2.687	<0.001
Social isolation	1.215	1.036–1.426	0.017
Experienced need for seeking health care	1.951	1.702–2.237	<0.001
Age	0.844	0.815–0.874	<0.001
Heterosexual	1		
Gay/lesbian	0.867	0.623–1.207	0.398
Bisexual	1.039	0.751–1.438	0.817
Other sexual orientation	1.372	0.905–2.079	0.136

*Full adjusted analysis controlling for gender (male vs. female gender). N = 7,967.*

### Result Gaming

Including all seven countries (*n* = 10 983) the prevalence of gaming problems in this study was 9.9% [*n* = 626, median 8.0 (IQR 7–13)] among the women and 13.8% [*n* = 765, median 9.0 (IQR 7–13)] in the male population with a statistical significance between the groups (*p* < 0.001). The largest proportion of problematic gaming behavior was found among individuals aged 15–18 years (25.4%, *n* = 99). In descriptive analysis, problematic gaming behavior was associated with loneliness (14.6 vs. 26.3% in the problematic gaming-group, *p* < 0.001), psychological distress (20.3 vs. 38.6% in the problematic gaming-group, *p* < 0.001), gender (11.4% among women vs. 13.8% among men, *p* < 0.001) and having a sexual minority status (12.2% reached the threshold for a problematic gaming behavior in the heterosexual group vs. 18.8% in the minority group, *p* < 0.001).

Among women, those with a problematic gaming behavior were significantly more likely to have experienced loneliness (15.6 vs. 28.3% in the problematic gaming-group, *p* < 0.00) and have a sexual minority status (10.7 vs. 20.6% in the minority group, *p* < 0.001). The largest proportion of women with a problematic gaming behavior was found among those aged 19–24 years (16.0%, *n* = 83) in the heterosexual group and in the minority group among 15–18 years old (37.2%, *n* = 42). In logistic regression analysis, age, social isolation, psychological distress and other sexual orientation remained significant (see Table 5).

**TABLE 5:** Problematic gaming women (*n* = 5,448).

	Odds ratio (OR)	Confidence interval (CI)	*p*-value
Sweden	1		
Denmark	0.635	0.391–1.031	0.066
England	3.416	2.393–4.876	<0.001
Italy	3.310	2.272–4.820	<0.001
Spain	4.987	3.504–7.097	<0.001
Poland	3.905	2.744–5.558	<0.001
Switzerland	1.386	0.927–2.073	0.111
Social isolation	1.611	1.308–1.983	<0.001
Experienced need for seeking health care	2.184	1.807–2.640	<0.001
Age	0.817	0.776–0.860	<0.001
Heterosexual	1		
Gay/lesbian	1.383	0.770–2.487	0.278
Bisexual	1.377	0.930–2.039	0.110
Other sexual orientation	2.323	1.367–3.946	0.001

In the male population, those with a problematic gaming behavior were significantly more likely to have experienced loneliness (13.6 vs. 24.6% in the problematic gaming- group, *p* < 0.001), psychological distress (15.0 vs. 35.6% in the problematic gaming- group, *p* < 0.001) and have a sexual minority status (13.6 vs. 17.1% in the sexual minority group, *p* = 0.033). The age group where a problematic gaming behavior was most common, was among 15–18 years in both the heterosexual (37.0%, *n* = 44) and minority group (28.6%, *n* = 4). Applying binary regression analysis, sexual minority status was not significantly associated with problem gaming in women (Table 5) or men (Table 6). In a full adjusted analysis including both women and men, problem gaming remained significantly associated with social isolation, need to seek treatment, male gender and with reporting the sexual minority status “other,” but not with other sexual minority identifications (Table 7).

**TABLE 6:** Problematic gaming men (*n* = 5,519).

	Odds ratio (OR)	Confidence interval (CI)	*p*-value
Sweden	1		
Denmark	0.779	0.493–1.229	0.283
England	4.572	3.172–6.590	<0.001
Italy	4.391	3.031–6.360	<0.001
Spain	4.685	3.246–6.763	<0.001
Poland	3.343	2.310–4.837	<0.001
Switzerland	1.285	0.839–1.969	0.249
Social isolation	1.554	1.261–1.915	<0.001
Experienced need for seeking health care	2.785	2.305–3.366	<0.001
Age	0.668	0.635–0.704	<0.001
Heterosexual	1		
Gay/lesbian	0.727	0.483–1.094	0.126
Bisexual	1.265	0.750–2.136	0.378
Other sexual orientation	1.318	0.679–2.561	0.415

**TABLE 7:** Variables potentially associated with problem gaming.

	Odds ratio (OR)	Confidence interval (CI)	*p*-value
Sweden	1		
Denmark	0.745	0.536–1.035	0.079
England	4.165	3.233–5.366	<0.001
Italy	5.135	3.988–6.611	<0.001
Spain	3.810	2.956–4.910	<0.001
Poland	1.357	1.013–1.817	0.041
Switzerland	1.428	0.671–3.039	0.355
Male gender	1.574	1.392–1.779	<0.001
Social isolation	1.562	1.349–1.809	<0.001
Experienced need for seeking health care	2.453	2.147–2.803	<0.001
Age	2.431	0.894–6.611	0.082
Sexual orientation			
Heterosexual	1		
Gay/lesbian	0.902	0.645–1.260	0.544
Bisexual	1.236	0.905–1.688	0.184
Other sexual orientation	1.810	1.196–2.738	0.005

*Full adjusted analysis controlling for gender (male vs. female gender). N = 10,967.*

### Result Problematic Internet Behavior

Including all seven countries (*n* = 10 983), 3.9% (*n* = 212) of the women and 3.5% (*n* = 191) of the men reached the threshold for having a problematic internet behavior. No statistical significance was found between the groups (*p* = 0.125). The median value was 2 in both groups, [(IQR 0–5) among women and (IQR 0–4) among men]. The largest proportion of problematic internet behavior was found among those aged 15–18 years old (9.3%, *n* = 36). In the whole group, a problematic internet behavior was associated with loneliness (15.3 vs. 37.5% in the group with a problematic internet behavior, *p* < 0.001), psychological distress (21.7 vs. 47.6% in the group with a problematic internet behavior, *p* < 0.001) and sexual orientation (3.5 vs. 6.3% in the minority group, *p* < 0.001).

In the female population, those with a problematic internet behavior were significantly more likely to have experienced loneliness (16.2 vs. 38.7% in the group with a problematic internet behavior, *p* < 0.001), have experienced psychological distress (26.6 vs. 46.7%, *p* < 0.001) and have a sexual minority status (3.7 vs. 6.8% in the sexual minority group fulfilled criteria, *p* = 0.004). In the binary regression analysis, having too few social contacts, psychological distress remained significant.

Among men, problematic internet behavior was associated with having too few social contacts and experience loneliness (14.4 vs. 36.1% in the group with a problematic internet behavior, *p* < 0.001), psychological distress 16.8 vs. 48.7% in the group with a problematic internet behavior, *p* < 0.001) and having a sexual minority status (3.3% fulfilled criteria for a problematic internet behavior in the heterosexual group vs. 5.9% in the minority group, *p* = 0.006). In the binary regression analysis, sexual minority status was not associated with problematic internet use in women (Table 8) or men (Table 9). When including both women and men in a full, adjusted analysis, problematic internet behaviors was significantly associated with younger age, social isolation, and with need to seek treatment, but not with gender or sexual minority status (Table 10).

**TABLE 8:** Problematic internet use women (*n* = 5,448).

	Odds ratio (OR)	Confidence interval (CI)	*p*-value
Sweden	1		
Denmark	0.550	0.238–1.274	0.163
England	2.659	1.503–4.704	0.001
Italy	3.171	1.752–5.739	<0.001
Spain	4.483	2.571–7.815	<0.001
Poland	3.738	2.150–6.500	<0.001
Switzerland	0.842	0.402–1.764	0.649
Social isolation	2.435	1.791–3.310	<0.001
Experienced need for seeking health care	2.153	1.593–2.911	<0.001
Age	0.770	0.708–0.837	<0.001
Heterosexual	1		
Gay/lesbian	1.590	0.669–3.780	0.294
Bisexual	1.132	0.623–2.058	0.683
Other sexual orientation	1.011	0.386–2.643	0.983

**TABLE 9:** Problematic internet use men (*n* = 5,519).

	Odds ratio (OR)	Confidence interval (CI)	*p*-value
Sweden	1		
Denmark	0.558	0.227–1.370	0.203
England	3.392	1.792–6.422	<0.001
Italy	2.673	1.358–5.260	0.004
Spain	2.795	1.432–5.454	0.003
Poland	3.385	1.790–6.403	<0.001
Switzerland	1.373	0.647–2.916	0.409
Social isolation	2.244	1.615–3.118	<0.001
Experienced need for seeking health care	3.472	2.540–4.745	<0.001
Age	0.760	0.694–0.833	<0.001
Heterosexual	1		
Gay/lesbian	1.235	0.672–2.268	0.497
Bisexual	1.102	0.455–2.666	0.830
Other sexual orientation	2.134	0.814–5.593	0.123

**TABLE 10:** Variables potentially associated with problematic internet behavior.

	Odds ratio (OR)	Confidence interval (CI)	*p*-value
Sweden	1		
Denmark	0.569	0.309–1.047	0.070
England	3.089	2.026–4.709	<0.001
Italy	3.010	1.932–4.690	<0.001
Spain	3.725	2.434–5.700	<0.001
Poland	3.724	2.458–5.641	<0.001
Switzerland	1.103	0.656–1.856	0.711
Male gender	1.071	0.869–1.319	0.519
Social isolation	2.345	1.876–2.932	<0.001
Experienced need for seeking health care	2.684	2.161–3.333	<0.001
Age	0.771	0.725–0.819	<0.001
Heterosexual	1		
Gay/lesbian	1.362	0.830–2.235	0.221
Bisexual	1.118	0.684–1.828	0.655
Other sexual orientation	1.442	0.730–2.848	0.292

*Full adjusted analysis controlling for gender (male vs. female gender). N = 10,967.*

## Discussion

The present study did not reveal independent associations between belonging to a sexual minority and screening positive for a problem gambling, problem gaming or problematic internet use behavior. Thus, this study could not confirm the hypothesis of an over-representation of behavioral addiction in sexual minority groups when controlling for a number of demographic and psycho-social variables. However, screening positive was more common in individuals belonging to a sexual minority, both with respect to gaming, problem internet use, and (for women) problem gambling. Thus, based on the present study, there is reason to highlight behavioral addictions in sexual minority group, although it is likely that their over-representation is due to the influence of co-factors.

Problematic internet behavior did not differ between women and men. All three behavioral addictions were significantly overrepresented in the sexual minority group in descriptive analyses, problematic gambling, gaming and internet use. The differences were greater between minority women and women with a heterosexual identity, compared to among men. In binary regression analysis, sexual orientation status was not significantly associated with excessive gambling, gaming or internet behavior when controlling for nationality, age, psychological distress and social isolation among men. Among women, having a sexual minority status in general was significantly associated with gambling and gaming behavior when controlling for age and nationality. When also controlling for psychological distress, social isolation and dividing sexual orientation into gay/lesbian, bisexual and other, only the minority group defining as other remained statistically significant for having a problematic gaming behavior among women.

In descriptive analyses, reaching the threshold for having an excessive gambling, gaming or internet behavior was positively associated with social isolation and psychological distress in the whole study population as well as within each gender (female and male). Problematic gambling and gaming behavior were overrepresented among men in the whole study population, in concordance with earlier publications (Abbott et al., 2014; Lemmens et al., 2015; Calado and Griffiths, 2016).

The result partially confirms the pilot study performed prior to this study, in a Swedish setting. The study revealed a higher proportion of a problematic gaming and internet behavior in the sexual minority population (Broman and Håkansson, 2018). Understanding the impact of gaming habits and psychological health and wellbeing is complex. While a low usage of electronical games has been associated as a protective factor against substance use, a problematic gaming behavior has inversely been associated with a higher substance use of nicotine, alcohol and cannabis along with ADHD, depression and anxiety (Vadlin et al., 2016; Feng et al., 2017; van Rooij et al., 2017; Yen et al., 2017; Turel and Bechara, 2019). Seeing that the field of behavioral addictions have shown an association with problems establishing meaningful relations outside of internet (Peters and Malesky, 2008; van Rooij et al., 2014; Laconi et al., 2017), minority populations with a higher risk for encountering discriminating behavior could find the online arenas attracting.

There is reason to further highlight and examine significance of problematic internet behavior. While this construct describes a maladaptive behavior with respect to internet itself, it does not specify the content of online behavior and thereby also not the specific consequences and correlates of specific actions and experiences made online. Scoring about the cut-off for a problematic online behavior may, therefore, reflect behavioral patterns which are addictive disorders or which constitute other maladaptive behaviors *per se* (Griffiths, 2020). It also has been highlighted by researchers that for an addictive internet behavior, it may be difficult to establish a clear-cut difference between an extensive leisure activity and an addictive disorder (Ng and Wiemer-Hastings, 2005). It is also possible that this may contribute to the sometimes wide range of prevalence figures reported for this condition (Mihara and Higuchi, 2017; Dahl and Helmersson Bergmark, 2020). The construct of problematic internet use, in the present study, was not associated with sexual minority status in the adjusted analyses, but was markedly more common in unadjusted analyses in sexual minorities as well as in individuals with poor mental health. Thus, altogether, there is reason for further study of online behavior in a broader sense than gambling for money or typical video-gaming, and to further outline, in future study designs, which components make up this potentially diagnostic construct.

In those few earlier publications that exist in behavioral addiction and sexual identity and behavior, gambling disorder was more prevalent among gay and bisexual men in an Northern American setting (Grant and Potenza, 2006) and in a Canadian study sample of college-students, where gambling problems were more prevalent among both sexual minority men and women (Richard et al., 2019). A possible explanation is that there might be differences between American and Canadian settings, in comparison to the European context in this survey. In the Canadian study, the specific study population could make it difficult to compare with this survey that was performed in a broader study sample. Seeing that several mental health problems have been overrepresented among both lesbian, gay and bisexual men and women, it is of importance to highlight if there are no significant differences in this study between gay and bisexual men, in comparison to heterosexual men for behavioral addictions.

Further, another finding was a statistical significance for having experienced psychological distress among those with a gaming problem and sexual minority status, in comparison to the heterosexual participants with gaming problems (*n* = 1,389, 52.4% in the minority group vs. 37%, *p* < 0.001). Psychological distress partially explained the variations in gambling and gaming problems in this study among sexual minority women, in adjusted analysis. Earlier research, even if the number of studies is limited within the field, have indicated a higher proportion of self-reported psychiatric symptoms among those with a gaming disorder (Vadlin et al., 2016; Wang H. R. et al., 2018). Although, in a Norwegian study with clinical interviews, a problematic gaming behavior among adolescents was not strongly associated with psychiatric comorbidity (Stenseng et al., 2019). Studies among LGBT-youth have revealed higher rates of depressive symptoms and disorders, anxiety and suicide attempts than among heterosexual populations (Lucassen et al., 2017; Di Giacomo et al., 2018). In this study there is no further assessment of psychiatric symptoms, limiting the opportunity to examine whether mental health problems could explain the prevalence of gambling and gaming problems among the participants in this study.

Women with a gambling disorder in the general population have been found to have a higher level of psychiatric comorbidity than men (Potenza et al., 2001; Grant et al., 2012; Håkansson et al., 2018). It is an indication that women with gambling problems have other characteristics compared with men, and with possible greater psychiatric needs. Since gambling disorder is overrepresented among men globally (Wong et al., 2003; Abbott et al., 2014; Del Pino-Gutiérrez et al., 2017), there is a risk of underrepresentation in female perspectives in both research and treatment facilities. Gender aspects on gambling disorder have been increasingly addressed in recent years, pointing out the importance of addressing gambling with a gendered approach (Bowden-Jones and Prever, 2017). If treatment facilities are based upon the knowledge from gambling patterns typically occurring in men, it could possibly affect the treatment availability for women with gambling disorder. For individuals with gambling or gaming problems that are both female and have a sexual minority status, the treatment availability might be an even greater challenge. The result in this study highlights the need of including sexual minority perspectives of women in further research and treatment facilities in behavioral addiction.

Sexual minority women have been associated with higher rates of depressive symptoms (Lucassen et al., 2017), possibly explaining the higher prevalence of gambling and gaming problems among bisexual women and women with other sexual orientation in this study. Among sexual minority women in this study, both having experienced psychological distress and social isolation explained the variation in problematic gambling behavior. It is suggested that anxiety problems, depression, and substance use problems are more common for bisexual populations in comparison to homo- and heterosexual individuals and specifically among bisexual women, and partially explained by victimization and stigma (Hequembourg and Brallier, 2009; Björkenstam et al., 2017; Bränström and Pachankis, 2018; Charlton et al., 2018; Johns et al., 2018). These factors could potentially explain a greater interest for online platforms such as gaming. Although, as described above, no assessment of either psychiatric comorbidity, experiences of stigma or victimization were performed in this study. Earlier studies have also indicated difficulties for lesbian and bisexual women gaining access to health care (Corliss et al., 2010; Munson and Cook, 2016).

Knowing that stigma is affecting the health in sexual minority populations to a greater extent than in heterosexual populations, it is reasonable to think that the online world is attractive for its anonymity and in search of relevant information (Lucassen et al., 2018) when having a sexual minority status. Hussein and Griffiths (2008) found that 57% of the participants in their study had engaged in gender swapping within games. It supports the hypothesis that the gaming world could be platform used explore sexual and gender identity, independent of the sexual or gender identity. There seems to be a complexity to the online world in a LGBT-perspective as online games also have been described to be typically hetero-normative (Shaw, 2009; Krobová et al., 2015). Sexism and homophobia is described in online chat forums in a report on gaming in a LGBT-perspective from the Swedish Federation for Lesbian, Gay, Bisexual, Transgender, Queer and Intersex Rights (Wennlund, 2014). This dualism may add to the complexity of sexual identification and gaming behavior. It brings up the question whether there is a greater risk for developing behavioral addictions, among lesbian, gay and bisexual populations. Gaming activities but to a certain degree also gambling with money, takes place in the electronical and online world (McCormack et al., 2013; Håkansson et al., 2017). Online activities have been described in qualitative research as a part of social isolation among young LGBT-individuals, when experiencing stigma from the surrounding society (Steinke et al., 2017). The result in this study, indicating a higher proportion of problematic gambling and gaming behavior among sexual minority women, raises the question whether sexual minority women engage in electronical activities as a way of coping with psychological distress, stigma and isolation.

### Implications for Future Research

The result in this study, indicating that there are no differences between sexual minority men and heterosexual men in behavioral addiction problems is of importance to highlight in the discussion about sexual minority health. Earlier research within the field of substance use has indicated similar results, whit less apparent differences in substance use between men with a heterosexual orientation and sexual minority men, in comparison to heterosexual and sexual minority women (Marshal et al., 2008; Coulter et al., 2018). A possibility is that there is a similar pattern for substance behavior and behavioral addiction among sexual minority men. In future research on behavioral addictions, it would be of value to include sexual orientation to further investigate whether minority women are overrepresented compared to heterosexual women, and investigate a possible correlation with substance use, other psychiatric conditions, social isolation and experiences of discrimination and stigma. More research is needed in other orientations than heterosexual, gay/lesbian and bisexual. For clinical settings treating behavioral addiction, an inclusion of the sexual minority perspective could be of importance, especially for sexual minority women. Researchers have underlined the lack of knowledge about the sexual minority women in clinical health care (Munson and Cook, 2016). A few LGBTQ-interventions have addressed coping mechanisms against stigma and minority stress in a psychiatric and psychological context (Pachankis et al., 2015; Lucassen et al., 2018) and could be a relevant alternative for clinical settings (Wang K. et al., 2018) when treating behavioral addiction. Treatment interventions via internet have been suggested to be a way of including sexual and gender minority populations (Schwinn et al., 2015; Steinke et al., 2017). If the online world is attracting bisexual individuals due to a decreased risk of discrimination and stigma, it could be of importance for clinicians to also consider it an opportunity to study online treatments within sexual and gender minority populations in clinical research projects (Schwinn et al., 2015; Steinke et al., 2017).

In future research on behavioral addictions, it would be of value to include sexual orientation to further investigate whether minority women are overrepresented compared to heterosexual women, and investigate a possible correlation with substance use, other psychiatric conditions and experiences of discriminating behavior. It is of importance to be able to differentiate between different sexual minority groups in future studies, seeing that living conditions could differ between the groups and influence the risk for excessive gambling or gaming. Qualitative studies would be of value to better understand the meaning of online activities such as gambling and gaming among sexual minority women. In addition, it can be argued that a screening tool used for problematic internet behavior does not provide in-depth descriptions about the nature of that internet behavior, and that the nature and content of such online behavior is likely to differ between women and men, and potentially across sexual identifications. Thus, future studies should further address how a potentially problematic online behavior may differ between groups with separate sexual or gender identifications.

In addition, future research should assess a more in-depth picture of psycho-social situation as a co-factor in studies of the association between behavioral addictions and gender and sexual identity. For example, gambling behaviors are closely associated with socio-economic and social situation, such as indebtedness and private financial situation. While such data cannot be interpreted from the present data, such variables are of value to study in the future.

### Limitations

Sexual minority populations are groups with a large heterogeneity and living conditions for LGBTQ-individuals are probable to differ in the included countries in this study. These living conditions includes social and economic factors that reasonably have different impacts on psychological health and mental health problems, although not controlled for in this study. On the other hand, country affiliation is controlled for in this study. This study had a cross-sectional study design and to draw further conclusions on behavioral addiction, studies with a longitudinal design are needed where sexual orientation status is included. The web survey design, even if cooperating with the company providing web panels, could have attracted individuals answering with an interest in gambling and gaming behavior to a higher extent than in the general population. It could have contributed to the high prevalence of problematic behavior in this study. The anonymous web-survey design applied in the study could include a risk of individuals responding more than once on the survey. In an earlier web-survey in a Swedish setting, no more than 0.7% of the answers were possible duplicates (Håkansson and Henzel, 2020). In a survey on gambling behavior in a Danish setting, 1.5% of the answers came from the same IP-address with possibility of being duplicates (Håkansson, 2021). These earlier studies indicate that duplicates in web-surveys are not common.

The gambling result in this study may have been affected by the exclusion of the Polish and Italian gambling result. In the Polish survey, the high proportion of problem gamblers might have been a result of a translation problem. Online surveys and surveys offering any form of compensation might be apprehended differently in the included settings, possibly affecting the high rates of problem gamblers in this study. Considering that the target of the survey was to examine excessive behaviors in minority populations which are already few, the exclusion of two countries could also have led to other effects on the result. It might conceal differences between the minority population and the heterosexual population, considering the limitation in number of participants with a minority status.

The acceptance for other orientations than heterosexual might differ between the included countries, which could have affected how people chose to define their orientation in the study. Although, the prevalence of sexual minorities in this study was varying between 6.7 and 8.7% in all included countries apart from Italy, where the prevalence was lower (4.3%). There is a lack of information about those defining as having *other* sexual orientation in this study. It limits the opportunity to draw any conclusions about the group in this study. It could be that they are a diverse group with individuals defining as asexual, pansexual or mostly heterosexual. Although, defining as pansexual or mostly heterosexual could still mean being attracted to others regardless of the sex (Fu et al., 2019) and might share similar features with those defining as bisexual. Further, in this study no differentiation was made for sexual or emotional attraction, as well as for sexual behavior.

## Conclusion

This study emphasizes the importance of including the sexual minority perspective in clinical settings, with a certain focus in sexual minority women. It is one of the first studies investigating behavioral addiction of gambling and gaming and how it presents in groups with different sexual orientation status, in a European setting. Associations were found among sexual minority women for problematic gambling and gaming behavior. Psychological distress and loneliness explained most of these associations, while a problematic gaming behavior was associated with those women defining as having another sexual orientation than hetero-, homo, or bisexual in adjusted analysis. More research is needed in problematic gambling and gaming behavior in different sexual minority populations with regard to psychiatric comorbidity and living conditions. An awareness of female sexual minority perspectives is relevant in facilities treating behavioral addiction.

## Data Availability Statement

The raw data supporting the conclusions of this article will be made available by the authors, without undue reservation.

## Ethics Statement

The studies involving human participants were reviewed and approved by the Swedish Ethical Review Authority, from the Bioethics Committee of the Medical University of Silesia in Katowice, Poland, from the Ethics Committee from the Milan-Bicocca University, Milan, Italy, and from the Ethics Comittee of the Bellvitge University Hospital, L’Hospitalet de Llobregat, Spain. Written informed consent for participation was not provided by the participants’ legal guardians/next of kin because: The type of data that was collected in this study was anonymous and no biological samples were collected. Parental consent for participation in this type of study was not needed from the age of 15 in all countries but England and Poland where the age limit was 16. No data was collected from participants below that age in respective country.

## Author Contributions

NB and AH applied for the Swedish permission. FP and EG applied for the Italian ethical permission. SJ-M applied for the Spanish approval. AS applied for the Polish permission. NB and AH were responsible for data collection and statistical analysis. NB wrote the draft. All authors contributed with reviewing and changing the draft into the final version, participated in the interpretation of the result, approved the final version, and participated in the procedure of ethical approval from the ethical board in each country that required such permission.

## Conflict of Interest

The authors declare that the research was conducted in the absence of any commercial or financial relationships that could be construed as a potential conflict of interest.

## Publisher’s Note

All claims expressed in this article are solely those of the authors and do not necessarily represent those of their affiliated organizations, or those of the publisher, the editors and the reviewers. Any product that may be evaluated in this article, or claim that may be made by its manufacturer, is not guaranteed or endorsed by the publisher.

## Footnotes

1
https://userneeds.com/en/
